# 

**Published:** 1917-03-17

**Authors:** 


### CONTENTS.

PAGE

**What is Truth?** ... 469

**The Brain Worker and his Food** ... 470

**Hospital and Institutional News** ... 471

The Westminster Hospital Fire—Plain Speaking by the Lord Mayor of Manchester—Compulsory Medical Examination—Two Notable Victims of the War—Deaths of Two Devoted Institutional Workers—Legacies and the War Loan—The Revision of Medical Exemptions—Glamorgan's Help for the Welsh Medical School—Tribunals and Hospital Staffs—The Exclusion of Women from Hospital Boards—Leicestershire's Work for War Charities—Mr. Hargreaves Improves the Occasion—An Interesting Anniversary—The End of a Sore Point in Wales?—Thefts of Hospital Stores—Are Soldiers' Comforts to be Curtailed?—On Taking Risks in Philanthropy—Charity and Speculative Enterprise—A New Use for Legacies—The Welch Family and the Lancashire Infirmary—The Reward of Skill and Service—A Gold Collection—Economical Management in Cornwall—Rate Relief for War Charities—A "Roll of Honour" Hospital—The Conditional Donation—This Week's Drug Market.

**Venereal Diseases at Guy's Hospital** ... 477

The Working of the New Scheme.

**National Insurance Act Developments...** ... 478

The Report of the Unofficial Commission of Investigation.

**The Editor's Letter-Box** ... 480

Mr. Squealer the Rat-Catcher.
Poor-Law Nurses and the N.P.L.O.A.
The National Poor-Law Officers' Association.
Claim for Rebate of Spirit Duty.

**The Matrons' and Sisters' Department** ... 481

The Training of Probationers.
Round the Hospitals.
Nursing Questions in New Zealand—The First Co-operation of Nurses—The Endowment Fund of the College—Nursing Problems in Ireland—Registration: A Plan of Campaign—Coercion of Poor-Law Nurses Must Cease.
The Royal College of British Nursing.

**Infant Mortality** ... 484

Groping for a Safe Basis of Indubitable Fact.

**Some Nursing Problems in Ireland** ... 485

Registration: its Limitations and a Plan of Campaign.

**Parliament and the Profession** ... 486

**Royal Red Cross (Second Class)** ... iv

Awards for Valuable Services in Connection with the War.

**The Institutional Worker** .. ... (Suppl.) 1

The Service of Patients' Dinners.
Questions for March.
Enquiries and Answers.

